# Injury of ascending reticular activating system associated with delayed post-hypoxic leukoencephalopathy: a case report

**DOI:** 10.1186/s12883-017-0917-z

**Published:** 2017-07-19

**Authors:** Sung Ho Jang, Hyeok Gyu Kwon

**Affiliations:** 1 Department of Physical Medicine and Rehabilitation, College of Medicine, Yeungnam University, Gyeongsan, South Korea; 2 Department of Physical Therapy, College of Health Sciences, Catholic University of Pusan, 57 Oryundae-ro, Geumjeong-gu, Pusan, 46252 Republic of Korea

**Keywords:** Delayed post-hypoxic leukoencephalopathy, Hypoxic brain injury, Ascending reticular activating system, Consciousness, Diffusion tensor tractography

## Abstract

**Background:**

Delayed post-hypoxic leukoencephalopathy (DPHL) is a demyelinating syndrome characterized by neurological relapse after an initial recovery from hypoxic brain injury. We describe a patient with impaired consciousness following DPHL, concurrent with injury of the ascending reticular activating system (ARAS) shown using diffusion tensor tractography (DTT).

**Case presentation:**

A 50-year-old male patient was in a drowsy mental state after exposure to carbon monoxide (CO) for about ten hours. About a day after the CO exposure, his mental state recovered to an alert condition. However, his consciousness deteriorated to drowsy 24 days after the exposure and worsened to a semi-coma state at 26 days after onset. When he started rehabilitation six weeks after the CO exposure, he had impaired consciousness, with a Glasgow Coma Scale score of 8 and a Coma Recovery Scale-Revised score of 8. On 6-week DTT, decreased neural connectivity of the upper ARAS between the intralaminar thalamic nucleus and the cerebral cortex was observed in both frontal cortices, basal forebrains, basal ganglia and thalami. The lower dorsal ARAS was not reconstructed on the right side, and was thin on the left side. The lower ventral ARAS was not reconstructed on either side.

**Conclusions:**

Using DTT, we demonstrated injury of the ARAS in a patient with impaired consciousness following DPHL. Our result suggests that injury of the ARAS is a plausible pathogenetic mechanism of impaired consciousness in patients with DPHL.

## Background

Delayed post-hypoxic leukoencephalopathy (DPHL), a rare clinical condition, is a demyelinating syndrome characterized by neurological relapse after an initial recovery from hypoxic brain injury caused by carbon monoxide (CO) poisoning, overdose of drug, and myocardial infarction [1–3]. The majority of DPHL cases are associated with CO poisoning [4]. Lee and Marsden divided DPHL into two general clinical categories: parkinsonism (masked face, rigidity, tremor, dystonic posturing, agitation) and akinetic mutism (apathetic and developed functional bowel and minimal primitive responses to pain) [5–13]. However, very little is known about impaired consciousness following DPHL.

Hypoxic brain injury predominantly involves the gray matter. MRI is recognized as the most sensitive and common imaging tool for hypoxic brain injury [14]. In contrast, DPHL predominantly involves the white matter. Many studies have reported abnormality of the white matter including basal ganglia following DPHL using neuroimaging tools such as conventional MRI, diffusion weight imaging, and MR spectroscopy [5–13].

Recently developed diffusion tensor tractography (DTT), derived from diffusion tensor imaging (DTI), has the unique capability to estimate the neural tract in the white matter and is able to find the subtle or invisible neural injury by detection of characteristics of water diffusion [15]. Injury of the ascending reticular activating system (ARAS), which is responsible for consciousness, has been reported in patients with hypoxic brain injury [16, 17]. However, no study of injury of the ARAS in patients with DPHL has been reported.

In this study, using DTT, we report on a patient with impaired consciousness concurrent with injury of the ARAS following DPHL.

## Case presentation

**Fig. 1:**
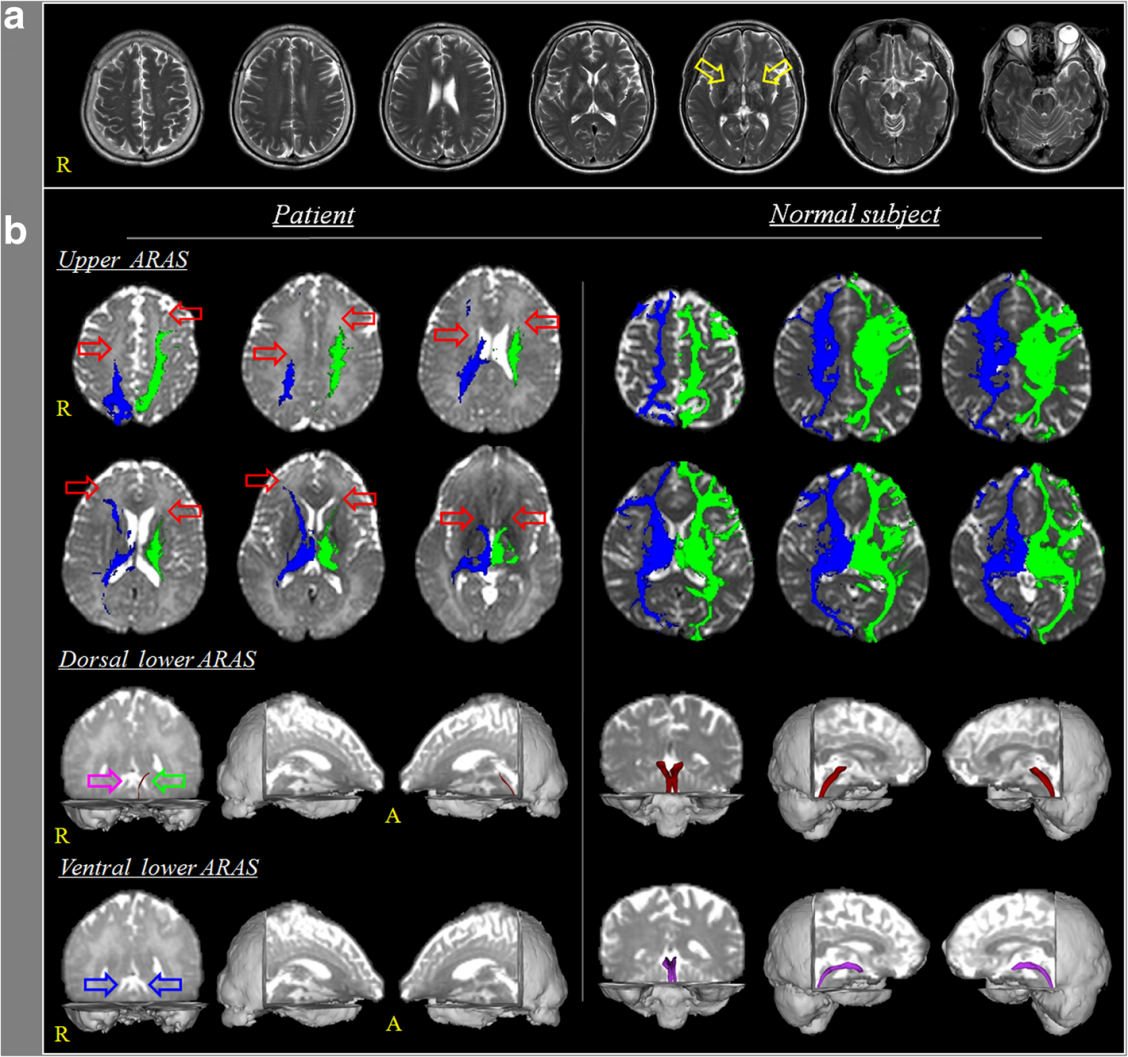
**a** Brain MR images at three weeks after onset show lesions in both basal ganglia (*yellow arrows*). **b** Results of diffusion tensor tractography (DTT) for the ascending reticular activation system (ARAS). On 6-week DTT, decreased neural connectivity of the upper ARAS between the intralaminar thalamic nucleus and the cerebral cortex is observed in both frontal cortices, basal forebrains, basal ganglia and thalami (*red arrows*). The dorsal lower ARAS between the pontine reticular formation and the intralaminar thalamic nucleus is not reconstructed on the right side (*purple arrow*) and thinning on the left side (*green arrow*). The ventral lower ARAS between the pontine reticular formation and the hypothalamus is not reconstructed on both sides (*blue arrows*). Results of DTT for the ARAS in a normal subject (53 year-old male). ARAS: ascending reticular activation system

A 50-year-old male patient showed drowsy mental state after exposure to carbon monoxide released from a coal briquette stove for about ten hours while he was sleeping. He underwent conservative management at a local hospital and his drowsy mental state recovered to an alert state approximately one day later without any neurological sequelae. However, he was transferred to the nephrology department of a university hospital for management of an acute kidney injury due to rhabdomyolysis ten days later. At that time, his Glasgow Coma Scale (GCS) and mini-mental state examination were full scores (15 and 30 scores, respectively) and results of blood test were as follows: creatine phosphokinase - 3273 IU/L (57 ~ 374), blood urea nitrogen - 133 mg/dL (8 ~ 23), creatinine - 5.67 mg/dL (0.6 ~ 1.5), aspartate aminotransferase - 53 IU/L (10 ~ 35), and alanine aminotransferase - 2 IU/L (0 ~ 40). Deep second degree contact burn wound was observed on his left buttock and he was diagnosed as a rhabdomyolysis which was caused by the contact burn. We assumed that the contact burn was occurred by the contact with the briquette stove during sleeping because there was no observer. At 16 days after the CO exposure, he began to show mild dysarthria and myoclonus on the right fingers. He developed clumsy movement 22 days after onset. His consciousness deteriorated to a drowsy state 24 days after onset and worsened to a semi-coma state at 26 days after onset. Brain MR images at three weeks after onset showed lesions in both basal ganglia (Fig. 1a). Six weeks after the CO poisoning, he was transferred to the rehabilitation department of the same university hospital. The patient showed impaired consciousness, with a Glasgow Coma Scale score of 8 (eye opening: 4, best verbal response: 1, and best motor response: 3) and a Coma Recovery Scale-Revised score of 8 (auditory function: 0, visual function: 3, motor function: 2, verbal function: 1, communication: 0, and arousal: 2) [18, 19]. The patient’s wife provided signed, informed consent, and the study protocol was approved by our Institutional Review Board.

## Magnetic resonance imaging and diffusion tensor imaging

Imaging parameters for T2-weighted MRI were as follows: acquisition matrix = 265 × 224, field of view = 210 × 210 mm^2^, repetition time = 4224.1 ms, echo time = 100 ms, number of excitations = 2, and a slice thickness of 5 mm with a gap of 2.2 mm. DTI data were acquired at six weeks after onset using a six-channel head coil on a 1.5 T Philips Gyroscan Intera with single-shot echo-planar imaging. Imaging parameters were as follows: acquisition matrix = 96 × 96; reconstructed to matrix = 192 × 192; field of view = 240 × 240 mm^2^; repetition time = 10,398 ms; echo time = 72 ms; echo-planar imaging factor = 59; b = 1000s/mm^2^; and a slice thickness = 2.5 mm. Affine multi-scale two-dimensional registration at the Oxford Centre for Functional Magnetic Resonance Imaging of Brain (FMRIB) Software Library was used to correct head motion effect and image distortion. Fiber tracking used FMRIB Diffusion (5000 streamline samples, 0.5 mm step lengths, curvature thresholds = 0.2), a probabilistic tractography method [20]. Three portions of the ARAS were reconstructed by selection of fibers passing through region of interest (ROI) as follows [21–23]: the upper ARAS, in which the neural connectivity of the intralaminar thalamic nucleus (ILN, ROI 1) to the cerebral cortex was analyzed, the dorsal lower ARAS, between the pontine reticular formation (RF, ROI 1) and the ILN (ROI 2), and the ventral lower ARAS, between the pontine RF (ROI 1) and the hypothalamus (ROI 2). Out of 5000 samples generated from the seed voxel, results for fiber tracking were applied at a threshold of two streamlines for the dorsal and ventral lower ARAS and 10 streamlines for the upper ARAS.

On 6-week DTT, decreased neural connectivity of the upper ARAS between the ILN and the cerebral cortex was observed in both frontal cortices, basal forebrains, basal ganglia and thalami (Fig. 1). The dorsal lower ARAS between the pontine RF and the ILN was not reconstructed on the right side and thin on the left side. The ventral lower ARAS between the pontine RF and the hypothalamus was not reconstructed on either side.

## Discussion and conclusions

In the current study, three portions of the ARAS (the dorsal lower ARAS, ventral lower ARAS and upper ARAS) in a patient with impaired consciousness following DPHL caused by CO poisoning were evaluated using DTT. We found that these three portions of the ARAS were injured in both hemispheres: the upper ARAS – decreased neural connectivity to both frontal cortexes, basal forebrains, basal ganglia and thalami, the dorsal lower ARAS – non-reconstruction in the right side and narrowing in the left side and the ventral lower ARAS –non-reconstruction in both sides. We believe that the impaired consciousness in this patient was ascribed to the injury of the three portions of the ARAS.

Many studies have reported abnormality of the white matter including basal ganglia (caudate nucleus, putamen, and globus pallidus) in patients with DPHL using various neuroimaging tools including conventional MRI [5–13]. Neurological manifestations were observed as follows: 1) cognitive impairments - confusion, disorientation, executive dysfunction, attention deficit, and akinetic mutism 2) motor symptoms - spasticity, hyper-reflexia, bradykinesia, rigidity, tremor, gait disturbance, dystonia 3) hallucinations, and 4) dysautonomia [5–13]. Regarding DTI, as far as we are aware, only one study was reported on patients with DPHL [24]. In 2008, Kenshi et al. demonstrated extensive white matter injury using DTI parameters (fractional anisotropy and mean diffusivity) in two patients with carbon monoxide intoxication (patient 1: frontal and parietal regions, globus pallidus, and corpus callosum and patient 2: globus pallidus) and showed neurological manifestations as follows: 1) patient 1 - akinetic mutism, disorientation, gait disturbance and 2) patient 2 - akinetic mutism [24]. To the best of our knowledge, this is the first DTT study to demonstrate injury of the ARAS in a patient with DPHL.

In conclusion, using DTT, we demonstrated injury of the ARAS in a patient with impaired consciousness following DPHL. Our result suggests injury of the ARAS is a plausible pathogenetic mechanism of impaired consciousness in patients with DPHL. However, because it is a single case report, this study is limited. In addition, several limitations of this study should be considered. First, use of DTT could lead to both false positive and negative results due to multiple fiber orientations in a voxel [25]. Second, we could not provide correlation between cognitive function and DTI anatomical site. Third, based on the blood test, we could not completely ruled out whether it affected neurogical status of the patient. Therefore, we suggest that further studies including large numbers of patients and overcoming limitations of this study should be encouraged.
